# Systematic Review of Free Tissue Transfer Used in Pediatric Lower Extremity Injuries

**Published:** 2021-03-08

**Authors:** Mehul Thakkar, Bartlomiej Bednarz

**Affiliations:** ^a^Department of Plastic and Reconstructive Surgery, St Mary's Hospital, London, United Kingdom; ^b^Department of Plastic and Reconstructive Surgery, Southmead Hospital, Bristol, United Kingdom

**Keywords:** free flaps, pediatric lower-limb reconstruction, pediatric lower-limb salvage, free tissue transfer, pediatric lower-limb trauma

## Abstract

**Aims:** To analyze the recent literature regarding the different types of free tissue transfer used in pediatric lower-limb trauma, trends, flap success rates, and the anatomical location of reconstruction. **Method:** A search was conducted involving the MEDLINE database using the key words “Paediatric,” “Pediatric,” “Children,” “Lower limb,” “Lower extremity,” “Leg,” “Ankle,” “Foot,” “Free flap,” “Flap,” “Microsurgery,” and “Free tissue transfer” in a 3-component search applying the Boolean operators “OR” and “AND.” The search was condensed to articles published in the last 5 years. **Results:** In total, 240 studies were retrieved. Thirty-nine titles were selected and after reviewing the abstracts, 10 articles fit the inclusion and exclusion criteria. A total of 220 free flaps were used to reconstruct defects. Age range was between 2 and 17 years. Complete flap failure rate was 4.5% (n = 10). The anterolateral thigh perforator flap was the commonest flap used (n = 59), and the latissimus dorsi flap was the commonest muscle flap used (n = 51). Sixty-five percent of flaps were fasciocutaneous/perforator, while muscle flaps accounted for only 33% of flaps. The foot and ankle region accounted for 72% of defects. **Conclusion:** With evidence of improved success rates, free tissue transfer has become a popular choice in reconstruction of pediatric lower-limb trauma injuries. This study shows that perforator/fasciocutaneous flaps have recently become a more popular choice over muscle flaps. Overall, the success rate of free flaps in pediatric lower-limb trauma is high (95.5%) and comparable with the adult population.

## SUMMARY

The aim of this review is to analyze the recently published literature regarding the different types of free tissue transfer used in pediatric lower-limb trauma, trends, flap success rates, and the anatomical location of reconstruction.

The advancement and high success rates of microsurgical free tissue transfer has have enabled large soft-tissue defects to be covered using a number of different types of autologous tissue. The ability to cover large soft-tissue defects with free tissue transfer has been extremely useful in limb salvage procedures resulting from trauma or even malignancies such as sarcoma.

Pediatric lower-limb trauma (PLLT) with extensive soft-tissue loss such as Gustilo-Anderson IIIB fractures poses a great challenge to the reconstructive surgeon due to concerns about vessel diameter size (both donor and recipient), increased vessel vasospasm potential, donor site availability, donor site morbidity, and long-term implications such as growth restriction and psychological impact. Despite these concerns, there is a growing consensus among reconstructive microsurgeons that pediatric free tissue transfer is a viable option with high success rates.^1^

The aim of this review was to analyze the recent published literature regarding the types of free tissue transfer used in PLLT, the recent trend, success rates, and the location of soft-tissue reconstruction in the lower limb.

## METHODS

The PRISMA guideline on systematic reviews was adhered to as much as possible.^2^ A literature search was conducted using the MEDLINE database through PubMed. The key words used were “Paediatric,” “Pediatric,” “Children,” “Lower limb,” “Lower extremity,” “Leg,” “Ankle,” “Foot,” “Free flap,” “Flap,” “Microsurgery,” and “Free tissue transfer” in a 3-component search using the Boolean operators “OR” and “AND.” The search was condensed to articles published in the last 5 years to include the most recent studies published. The titles were used to further narrow down the search, and abstracts of all subsequent studies retrieved were reviewed by the 2 authors; eligibility was determined using the following inclusion and exclusion criteria. Inclusion criteria included series of pediatric populations alone or where pediatric populations underwent subgroup analyses, case series of more than 2 patients, studies pertaining to soft-tissue coverage in traumatic lower-limb injuries using free tissue transfer among the cohort, and English language publications. Exclusion criteria included mixture of adult and pediatric population data with no pediatric subgroup analysis, single-series case reports, upper extremity reconstructions such as toe-to-digit transfers, and free tissue transfers for non–trauma-related lower-limb reconstructions.

## RESULTS

In total, 240 studies were retrieved from the search using the aforementioned key words. Thirty-nine titles were selected and after reviewing the abstracts, 10 articles fit the inclusion and exclusion criteria. Data extracted from the 10 relevant studies included number of patients, number of free flaps, age (mean/range), total complete flap failure rate, flap types, location of trauma, time period of data collection, country of institution where the study took place, and type of study. Levels of evidence were assigned to each study according to the Oxford levels of evidence 2.^3^ Results are shown in Tables 1-3.

Among the 10 studies included in the study, there were a total of 220 free flaps used to reconstruct traumatic lower-limb defects. The age range was between 2 and 17 years. The total complete flap failure rate was 4.5% (10/220). The anterolateral thigh (ALT) perforator flap was the commonest flap used (n = 59), and the latissimus dorsi (LD) flap was the commonest muscle flap used (n = 51). Sixty-five percent of flaps were fasciocutaneous/perforator, while muscle flaps accounted for 33% of flaps. The commonest area of reconstruction was the foot and ankle region, accounting for 72% (116) of defects (N = 161), as location of defect could not be extracted from 3 studies.

## DISCUSSION

As described previously, lower-limb trauma with corresponding soft-tissue defect poses a great challenge to the reconstructive surgeon in terms of decision making. Before any reconstructive procedures can be undertaken, a thorough initial evaluation of the patient should take place. Subsequent discussions should be had with the patient's parents and/or the child if old enough on the options available, the risks involved, and, most importantly, the lengthy rehabilitation process. The decision to amputate or proceed with limb salvage is a difficult one, and scoring systems such as the NISSSA (nerve injury, ischemia, soft-tissue injury, skeletal injury, shock, and age) score have been developed to aid clinicians in the decision-making process.^14^ Ideally, such decisions should be made in a center experienced with treating such injuries ideally with orthoplastic capabilities.

Prior to any reconstruction, initial surgical debridement of any devitalized tissue must be undertaken. Careful evaluation of the condition of underlying soft tissues, bones, nerves, tendons, ligaments, and vessels must be done. The scale of the reconstruction is then appreciated in terms of defect size, recipient vessel availability (size and length outside the zone of trauma), soft-tissue reconstruction required (skin coverage, nerve grafts, bone grafts, tendon reconstruction), and any orthopedic intervention required, with further planning required.^1^ The ultimate goal of such reconstructions is to restore form, function, and contour of the limb in question.^15^

Harii and Ohmori^16^ conducted the first free tissue transfers in the pediatric population in 1975, and Ohmori et al^17^ were able to successfully perform a groin flap in a 3-month-old child in 1977, leading to the advent of pediatric free tissue transfer. One of the limiting factors worrying microsurgeons in pediatric free tissue transfers is the diameter of vessels deemed to be safe for microsurgical anastomosis. Gilbert^18^ provided an arbitrary figure of 0.7 mm as the lower limit. However, vessel diameters of between 0.3 and 0.5 mm have been successfully anastomosed^19^^,^^20^ in the dawn of a new era of “super microsurgery.” It is recommended that more than 1 anastomosis is performed when very small vessels are encountered (<0.5 mm) to reduce the risk of failure.^21^

In a series of 106 pediatric free tissue transfers by Canales et al,^22^ the flap success rate was 88% and together with minor complication rates this was comparable with the adult population, paving the way for the use of free tissue transfer in the pediatric population. Indeed, with further experience over the last 3 decades in microsurgical soft-tissue reconstruction in children, high flap success rates of between 95% and 100% have been reported.^10^ In their systematic review of 439 flaps in pediatric lower-limb salvage following trauma, Jabir et al^21^ reported a 5.01% failure rate. This was comparable with our review of 220 free flaps where the overall success rate was 95.5% (10/220), strengthening the case.

Another important conundrum faced is the choice of flap used by the reconstructive surgeon. With a vast array of flaps available in the armamentarium, careful thought and planning are required on which flap will be of most benefit to reconstruct an individual defect. Factors that need to be considered are defect size, length/diameter of the pedicle/recipient vessels, and the need for any neurotization.^1^ Ultimately, a considerable amount of judgment and experience is required. The top 5 flaps used in this study included ALT flap, LD flap, scapular/parascapular flap, deep inferior epigastric artery perforator flap, and gracillis flap. The most popular flap was the ALT flap (27%), followed closely by the LD flap (23%). The majority of flaps were fasciocutaneous/perforator flaps, accounting for 65% of flaps in comparison with muscle flaps, which accounted for 33% of flaps. This is similar to the review by Claes et al,^15^ in which perforator/fasciocutaneous flaps were a more popular option, with the ALT flap being the most popular choice. Of note, in our study, 28 and 19 (47/51) LD flaps, respectively, were from 2 case series that took place over a period of 38 and 15 years, respectively. Similar to the studies of Claes et al^15^ and Jabir et al,^21^ this series points to a migration from the use of muscle flaps in earlier studies to the use of perforator-based fasciocutaneous flaps, with the latter becoming more popular.

Advantages of perforator flaps are that they are thin and pliable for reconstruction of the distal part of the leg (ankle, foot, heel, sole), large skin islands can be harvested (eg, thoracodorsal artery perforator flap) with reduced donor site morbidity due to direct closure, can be sensate if neurotized, can be debulked in the future, and have a reliant anatomy. Being thin and pliable are vital in reconstruction of the ankle, foot, and heel due to cosmetic appearance, range of motion required, and also ability to fit into footwear.^21^ In contrast, muscle flaps lead to functional loss at the donor site and additional skin grafting is required, further increasing donor site morbidity.^15^ Also, muscle flaps can create bulk to fill large dead spaces, add additional vascularity to the wound, and, in select cases, be used as a neurotized free functioning flaps.^15^

In this study, the distal third of the leg (ankle, foot, heel/sole) was the location of the majority of soft-tissue reconstructions (n = 116), followed by the lower leg (n = 42), which again mirrors the systematic review by Jabir et al^21^ across most series of pediatric free flaps for lower-limb injuries. This may be due to the fact that there is a shortage of local soft tissue in this mobile region, without much pliability and extensibility and possibly exposed tendons. The majority of pediatric lower-limb injuries are caused by road traffic accidents, and these predominantly involve the foot and ankle, accounting for the pattern described earlier.^23^^-^25 In the United States, approximately 9400 children are treated yearly for lawn mower injuries and 37% of these account for injuries to the lower extremity, feet, and toes.^26^ These make up the most extensive lower-limb injuries encountered in the United States.^15^

## LIMITATIONS

The study was limited to a single database, and only studies published over the last 5 years were included in order to give an idea of the recent trends. Titles and abstracts were reviewed by 2 authors, which may lead to bias or articles being missed. The retrieved studies are all retrospective case series with a level of evidence of 4.

In 3 studies, defect location was not extractable. Minor flap complications such as partial necrosis or flap salvage procedures were not taken into account and overall limb salvage rates were not extracted, which may give more beneficial functional outcomes.

Further categories such as defect size, follow-up duration, time to soft-tissue coverage, donor/recipient vessel diameters, individual flap-type failure rates, reason for flap failure, and Gustilo-Anderson classification of injuries were beyond the scope of this study but would add further insight into flap selection and successful limb salvage.

## CONCLUSION

With evidence of improved success rates, free tissue transfer has become a popular choice in reconstruction of pediatric lower extremity injuries. This study shows that perforator/fasciocutaneous flaps have recently become a more popular choice over muscle flaps. Overall success rate of free flaps is high (95.5%) and comparable with the adult population. The majority of flaps are used to reconstruct defects distal to ankle region, followed by the leg, indicating injury patterns and areas redundant to local/regional flap options. There is a plethora of flaps available to the reconstructive surgeon, and flap selection should be individually tailored depending on the skill, experience, and repertoire of the surgeon and the defect presented in order to ultimately achieve the final goal of restoring optimal function. With this in mind, future studies should take into account functional outcomes and patient-reported outcomes in order to paint a better picture of the success or failures of reconstruction in this population of patients.

## Figures and Tables

**Table 1:** Demographics of studies included

Study	No. of patients	No. of flaps	Age, mean (range), y	% Flap failure	Year range	Study type	Level of evidence	Country
Sui et al (2019)^4^	42	42	(2-14)	0	2004-2016	Retrospective case series	4	China
Khadim et al (2019)^5^	33	30	(5-16)	3 (1)	2011-2017	Retrospective case series	4	United Kingdom
Lee et al (2020)^6^	49	53	13	11.3 (6)	1979-2017	Retrospective case series	4	USA
Luo et al (2019)^7^	10 (1 UL)	9	5.9 (2-11)	0	2011-2016	Retrospective case series	4	China
Branch et al (2017)^8^	27	2	5.5	0	2005-2015	Retrospective case series	4	United States
Franco et al (2017)^9^	4	4	9 (5-13)	0	2001-2012	Retrospective case series	4	United States
Momeni et al (2017)^10^	40	40	11.4 (1-17)	5 (2)	1997-2012	Retrospective case series	4	United States
Hu et al (2015)^11^	25	25	8.3 (4.5-14)	0	2008-2013	Retrospective case series	4	China
Acar et al (2015)^12^	11	11	8.9 (3-15)	0	2010-2013	Retrospective case series	4	Turkey
Laine et al (2016)^13^	8	4	10.4 (4-15)	25 (1)	1990-2010	Retrospective case series	4	United States

**Table 2:** Types and number of free flaps used (N = 220)*

Flap type	Total
Fasciocutaneous	
ALT	59
Scapular/parascapular	44
DIEP	30
Radial forearm	4
Groin	3
Lateral arm	2
TDAP	1
Deltoid	1
Muscle	
Latissimus dorsi	51
Gracillis	13
Rectus abdominis	8
Serratus	1
TFL	1
Fascial	
Temporoparietal fascia	1
Bone	
Fibula	1

*ALT indicates anterolateral thigh; DIEP, deep inferior epigastric artery perforator; TDAP, thoracodorsal artery perforator; TFL, tensor fasciae latae.

**Table 3:** Location of trauma and subsequent soft-tissue reconstruction (N = 161)*

Defect location	Number of flaps
Thigh	2
Knee	1
Lower leg	42
Ankle	37
Foot and ankle	17
Dorsal foot	39
Heel/plantar	23

*Location not specified in 3 studies: Lee et al,^6^ Branch et al,^8^ and Franco et al.^9^
